# Macular neovascularisation subtype determines visual consequences of early fibro-atrophic remodelling in neovascular AMD: PRECISE study report 11

**DOI:** 10.1038/s41433-026-04858-7

**Published:** 2026-09-17

**Authors:** Livia Faes, Syed Kubravi, Kimberly Spooner, Sridevi Thottarath, Sobha Sivaprasad, Dun Jack Fu

**Affiliations:** 1 NIHR Biomedical Research Centre at Moorfields Eye Hospital NHS Foundation Trust, UCL Institute of Ophthalmology, London, UK; 2 Cantonal Hospital Winterthur, Winterthur, Switzerland; 3 Epidemiology, Biostatistics & Prevention Institute (EBPI), University of Zurich, Zurich, Switzerland; 4 Medical Retina and Uveitis Department, University Hospitals Bristol and Weston NHS Foundation Trust, Bristol, UK; 5 Save Sight Institute, The University of Sydney, Sydney, NSW Australia; 6 The Graduate School of Health, The University of Technology Sydney, Sydney, NSW Australia; 7 Bradford Teaching Hospital NHS Foundation Trust, Bradford, UK; 8 King’s College Hospital NHS Foundation Trust, London, UK; 9 Newcastle Hospitals NHS Foundation Trust, Newcastle, UK; 10 The Royal Liverpool and Broadgreen University Hospitals NHS Foundation Trust, Liverpool, UK; 11 Leeds Teaching Hospitals NHS Trust, Leeds, UK; 12 South Tyneside and Sunderland NHS Foundation Trust, Sunderland, UK; 13 Frimley Health NHS Foundation Trust, Surrey, UK; 14 James Paget University Teaching Hospitals NHS Foundation Trust, Norfolk, UK; 15 York Teaching Hospital NHS Foundation Trust, York, UK

**Keywords:** Prognostic markers, Macular degeneration

## Abstract

**Purpose:**

To evaluate whether baseline well-delineated hyperreflective material (wdHRM) is associated with short-term visual outcome in treatment-naïve neovascular age-related macular degeneration (nAMD), whether this association differs by macular neovascularisation (MNV) subtype, and how wdHRM relates to atrophy-related OCT biomarkers.

**Methods:**

Multicentre observational analysis of 2036 treatment-naïve eyes (PRECISE cohort) completing three monthly aflibercept 2 mg injections. Best-corrected visual acuity (BCVA) at Visit 4 (V4) was modelled against baseline wdHRM using adjusted multivariable linear regression. Secondary analyses assessed subtype interaction and eye-level association with atrophy-related OCT biomarkers.

**Results:**

Mean BCVA improved from 58.0 to 62.6 letters by V4. Baseline wdHRM was present in 14.6% of eyes, and foveal-involving wdHRM independently predicted lower V4 BCVA (β =-6.60 letters, 95%confidence interval [CI]-8.18 to -5.02; *p* < 0.001). Foveal-involving baseline choroidal hypertransmission (HTM) showed a stronger adverse association (β =-8.98, 95%CI-10.97 to -6.98; *p* < 0.001). The wdHRM association differed by subtype (interaction *p* = 0.016), with adjusted BCVA reductions of -10.6 letters in Type 3 MNV, -4.8 in Type 2/mixed MNV, -4.1 in Type 1 MNV, and -1.0 in polypoidal choroidal vasculopathy. By V4, wdHRM increased to 21.0% and was associated with HTM and greater outer retinal disruption.

**Conclusions:**

In treatment-naïve nAMD, baseline wdHRM is independently associated with poorer short-term visual outcome, particularly in Type 3 MNV. Short-term visual outcome should be interpreted in relation to both fibrosis-related and atrophy-related OCT biomarkers.

## Introduction

Fibrosis is one of the most formidable barriers to sustained visual recovery in exudative neovascular age‑related macular degeneration (nAMD), even in the era of anti‑angiogenic therapy [1]. Despite major therapeutic advances, fibrotic sequelae remain common, developing in up to one fifth of treated eyes by one year, approximately one third by two years, and in the majority by five years [1]. Importantly, about 60% of the 5‑year fibrotic burden accumulates within the first treatment year [1–8]. In real-world cohorts, macular fibrosis is consistently associated with substantially worse visual function at presentation and persistently poorer outcomes during follow-up, in a severity-dependent manner [1, 5, 9]. In those studies, patients with subretinal scarring were more likely to experience clinically meaningful visual acuity decline than eyes without fibrosis [1, 10].

Histopathologically, fibrosis in nAMD denotes excessive extracellular-matrix deposition, predominantly collagen, fibronectin and laminin, by myofibroblast-rich fibrovascular tissue within the subretinal or sub-RPE compartment [11, 12]. Although in vivo imaging is used to infer fibrosis, definitive identification remains challenging because operational criteria vary and tissue confirmation is impractical in clinical care [12].

Contemporary practice relies heavily on spectral-domain optical coherence tomography (SD-OCT) to characterise fibrosis-related change in nAMD. On OCT, fibrosis has often been inferred from subretinal or sub-RPE hyperreflective material (HRM), particularly when it appears homogeneous, well-delineated (wdHRM), and plaque-like. However, HRM is a descriptive OCT term rather than a histologic diagnosis and may include exudative or haemorrhagic material as well as more organised fibrovascular tissue [12–14]. Several groups have therefore distinguished ill-defined, heterogeneous (S)HRM from more sharply delineated wdHRM, the latter being more consistently associated with poorer visual outcomes [15–18]. Recent *International Fibrosis Consensus* recommendations likewise position SD-OCT as the primary screening modality for fibrosis, but emphasise its limited specificity in isolation and recommend multimodal confirmation for definite fibrosis in clinical studies [14]. Accordingly, in the present study, baseline HRM phenotypes were treated as a fibrosis-related biomarker rather than a definitive diagnosis of established fibrosis.

Increasing clinicoradiologic evidence indicates that fibrosis and atrophy are not mutually exclusive endpoints in treated nAMD but often co-localise, with residual HRM detectable within areas labelled as outer retinal or retinal pigment epithelium (RPE) atrophy. This fibro-atrophic continuum has recently been described as “atrosis” and may carry risks distinct from those of primary geographic atrophy in a non-neovascular context [19]. The heterogeneity in fibrosis propensity, atrophy and visual prognosis across MNV subtypes introduces further complexity. Observational and trial analyses converge on higher fibrosis risk in Type 2- or mixed lesions, lower rates in Type 3 MNV, and intermediate risk (and often sub‑RPE in phenotype) in Type 1 MNV (including polypoidal choroidal vasculopathy [PCV]) [1, 20, 21]. These subtype patterns imply that unstratified associations may be misleading and must be analysed with subtype‑specific context.

These considerations leave several clinically relevant uncertainties at treatment initiation in nAMD. It remains unclear whether baseline wdHRM, used here as an OCT-defined biomarker of fibrotic remodelling, independently predicts short-term visual outcome after induction therapy, whether its functional consequences differ by MNV subtype, and how it relates at eye level to atrophy-related OCT biomarkers during early treatment.

Using the PRECISE cohort, we addressed two related questions. First, we examined whether baseline wdHRM predicts BCVA after three monthly aflibercept injections, after adjustment for prespecified baseline and Visit 4 (V4) covariates. Second, we characterised the short-term evolution of HRM phenotypes and their eye-level association with atrophy-related OCT biomarkers by V4.

## Methods

### Study design and ethical considerations

This was a multicentre observational analysis within the PRECISE programme of treatment‑naïve nAMD eyes initiated on aflibercept 2 mg and assessed at baseline and after completion of three monthly injections (post‑“loading” or therapy induction, V4). The dataset taken forward for analysis comprised 2036 eyes with tracked baseline and V4 OCT and BCVA measurements drawn from the consolidated PRECISE data freeze used for this report. Patient recruitment occurred between December 2019 and August 2021 across United Kingdom centres with harmonised protocols. Aflibercept treatment schedules and visit timing that frame the present analysis have been published elsewhere. All procedures adhered to the Declaration of Helsinki with local institutional approvals and consent according to PRECISE governance as described in previous publications [22–28].

### Imaging acquisition and central reading

Participating sites acquired SD-OCT (*Spectralis; Heidelberg Engineering*) using fovea-centred raster scans ranging from 20° × 25° to 30° × 20°. A central reading workflow harmonised feature definitions across sites as per prior PRECISE reports [22–28]. OCT grading in PRECISE was performed using a predefined grading manual by trained medical retina fellows after calibration on a test set of 50 OCT images. Baseline imaging in the present analysis underwent double grading to enhance reliability, whereas V4 imaging was subject to single grading because of study logistics and resource constraints. Graders were masked to outcomes where applicable.

**Fig. 1:**
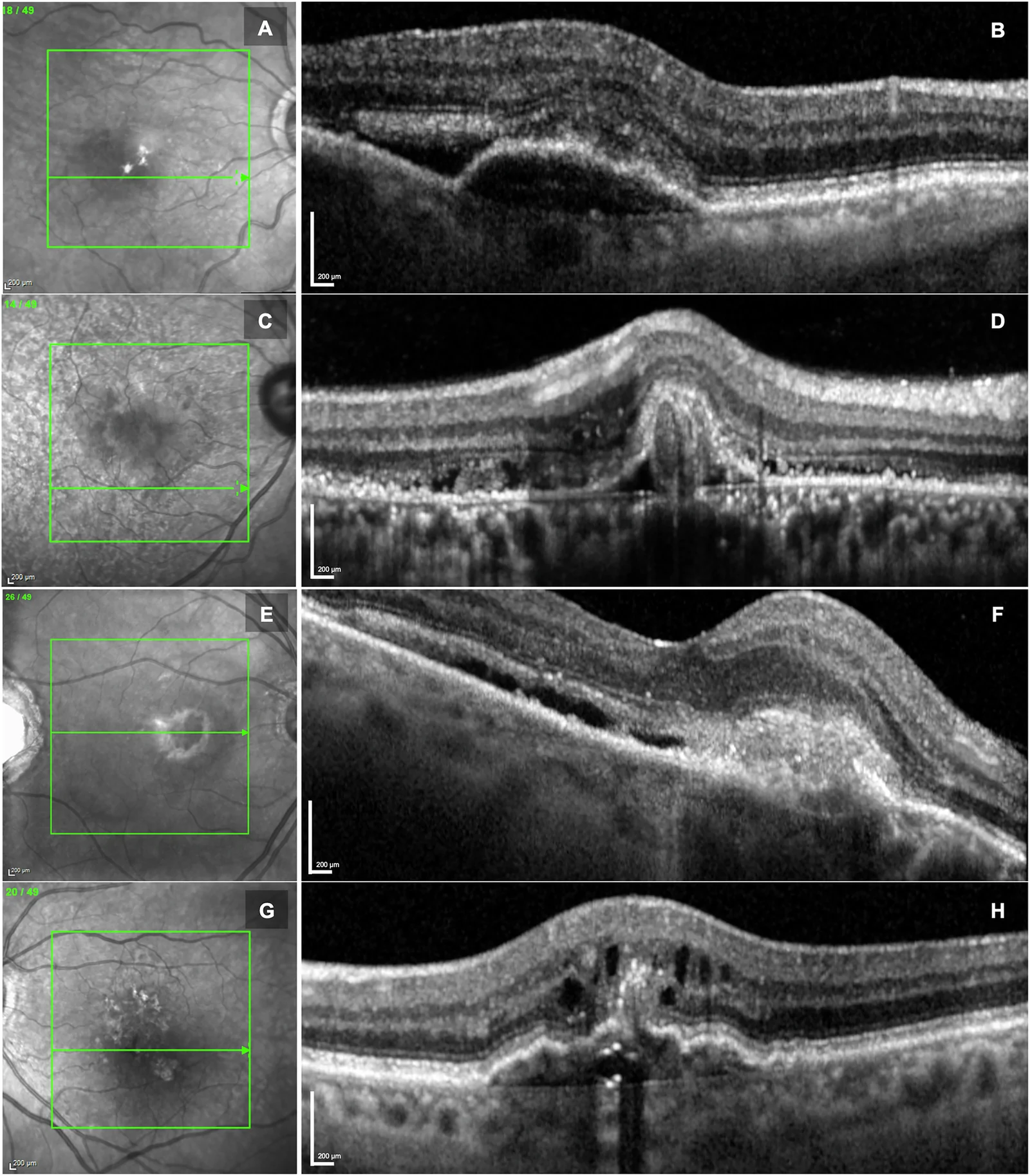
Illustrative Examples of Macular Neovascularisation Subtypes in Neovascular Age-Related Macular Degeneration Selected from The PRECISE Dataset. **A–H** All panels show multimodal imaging acquired with Spectralis spectral-domain optical coherence tomography (SD-OCT, *Heidelberg Engineering, Heidelberg, Germany*). **A C E** and **G** show near-infrared reflectance images of the macula, with the green line indicating the location of the corresponding horizontal SD-OCT B-scan. **B, D, F**, and **H** show the corresponding SD-OCT B-scans. **A, B** Example of type 1 macular neovascularisation. **C, D** Example of polypoidal choroidal vasculopathy, with a peaked pigment epithelial detachment and ring sign. **E, F** Example of type 2/mixed macular neovascularisation with subretinal hyperreflective material. **G, H** Example of stage 3 type 3 macular neovascularisation, with intraretinal fluid and a spheroid lipid globule in the sub-retinal pigment epithelium space producing a lensing effect.

Neovascular subtype (Type 1-, Type 2/mixed-, Type 3 MNV, and polypoidal choroidal vasculopathy [PCV]) was determined from structural OCT morphology. PCV was recorded where clinically diagnosed at the treating site. No systematic invasive angiography was undertaken. Figure 1 provides illustrative examples of MNV subtypes in PRECISE.

### Pre-specified operational definitions

#### Fibrosis-related OCT biomarkers

(S)HRM: (Subretinal) hyperreflective material on structural OCT located in the subretinal space, with reflectivity greater than adjacent fluid and surrounding outer retinal tissue, but with ill-defined borders and heterogeneous internal appearance, considered morphologically compatible with a mixed lesion containing exudative and/or fibrovascular components.

wdHRM: Hyperreflective material on structural OCT with dense, homogeneous internal reflectivity and sharply delineated anterior margins, with or without banded or laminated appearance. Where the reference was assessable on the same B-scan, reflectivity was typically comparable to or greater than adjacent normal RPE. In scans where normal RPE visibility was obscured by lesion architecture, classification relied additionally on morphologic criteria including homogeneity, sharp delineation, and lamination.

(S)HRM and wdHRM were adjudicated on structural OCT; colour fundus photography (CFP) and fluorescein angiography (FA) were not required as standardised inputs for this classification. This operational definition differs from the recent International Fibrosis Consensus (IFC) framework, which was developed for multimodal adjudication of definite fibrosis in clinical studies using SD-OCT together with confirmatory CFP or FA, typically at later post-treatment timepoints [14]. In the present study, baseline wdHRM in treatment-naïve eyes was used as an OCT-defined fibrosis-related biomarker of early fibro-atrophic remodelling rather than as a definitive multimodal diagnosis of established fibrosis.

#### Atrophy-related OCT biomarkers

**Fig. 2:**
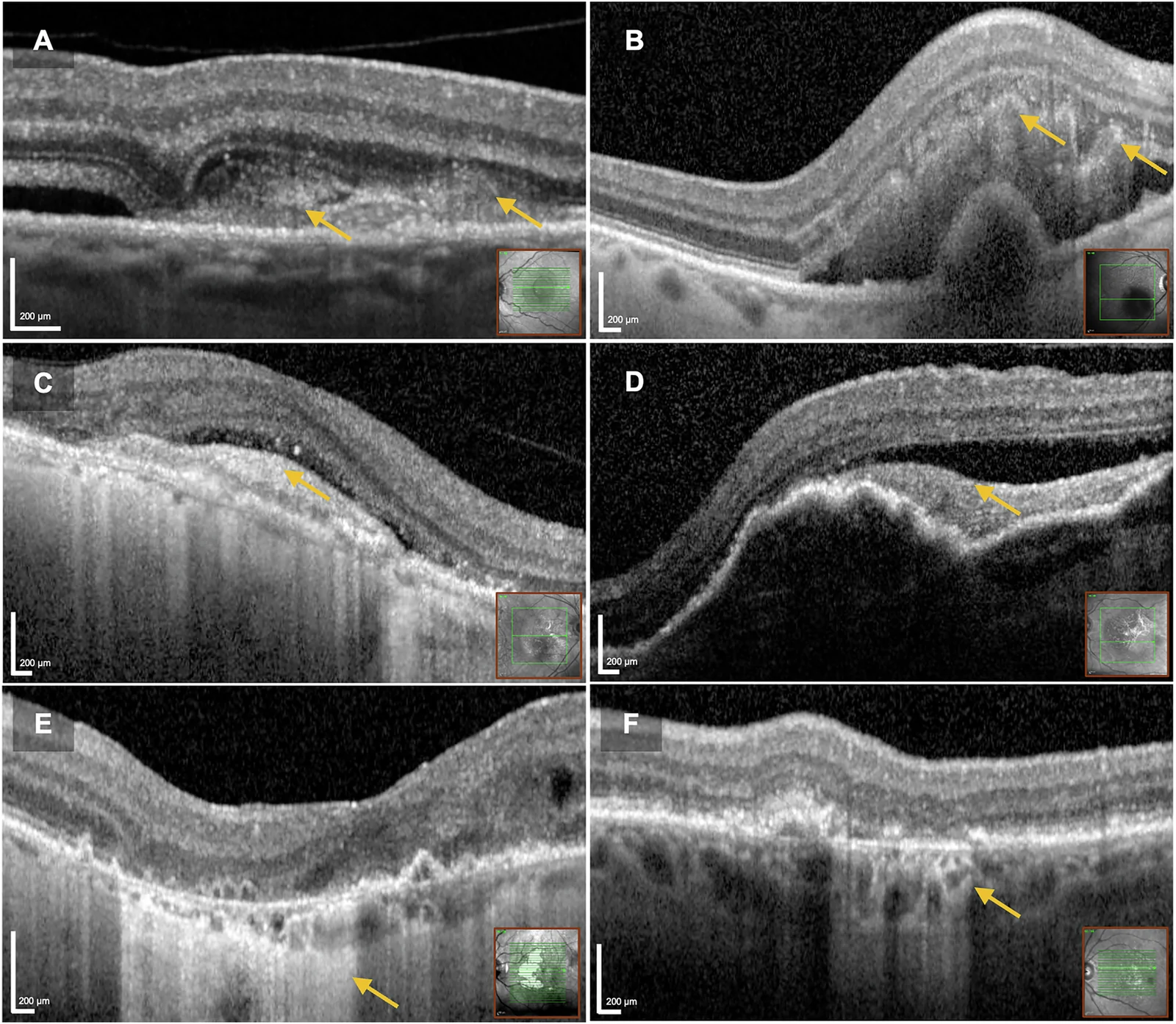
Illustrative Examples of Subretinal Hyperreflective Material, Well-Delineated Hyperreflective Material and Choroidal Hypertransmission in Neovascular Age-Related Macular Degeneration Selected from the PRECISE Dataset. **A–F** All panels show horizontal spectral-domain optical coherence tomography B-scans through the macula (SD-OCT, *Spectralis OCT; Heidelberg Engineering, Heidelberg, Germany*). The inset in each panel shows the corresponding near-infrared reflectance image, with the green line indicating the location of the OCT B-scan. **A, B** Examples of subretinal hyperreflective material (SHRM). The yellow arrows indicate the fuzzy, poorly delineated anterior border of the hyperreflective material. **C, D** Examples of well-delineated hyperreflective material (wdHRM). The yellow arrows indicate the sharply delineated anterior border of the lesion. Panel **D** also shows an impending retinal pigment epithelium tear. **E, F** Examples of choroidal hypertransmission (HTM). The yellow arrows indicate the border of the HTM.

Atrophy was not adjudicated as a formal lesion-level diagnosis in this study. Instead, atrophy-related remodelling was operationalised using OCT biomarkers, namely choroidal hypertransmission (HTM) and outer retinal structural disruption captured by ellipsoid zone (EZ)-loss and external limiting membrane (ELM)-loss. The present analysis focused on early fibro-atrophic remodelling rather than formal complete retinal pigment epithelium and outer retinal atrophy (RORA) adjudication. Therefore, HTM, EZ-, and ELM loss were analysed as separate biomarkers. EZ and ELM status were graded as present, absent, or ungradable where obscured by lesion burden or scan limitations. Dedicated *en face* lesion mapping or size quantification of HTM was not performed. Figure 2 provides illustrative examples of fibrosis- and atrophy-related OCT biomarkers.

Topographic definitions: Subfoveal was defined as directly beneath the central foveal point. Foveal involvement was defined as extension of a lesion or biomarker into the central 1 mm Early Treatment Diabetic Retinopathy Study (ETDRS) subfield. Extrafoveal or non-foveal was defined as a location entirely outside the central 1 mm region. Topographic co-localisation between wdHRM and EZ loss, ELM loss, or HTM was not encoded in PRECISE. Therefore, lesion-level spatial inference was not possible. For the primary prognostic model, HTM was selected as the atrophy-related covariate, whereas EZ- and ELM loss were reserved for secondary descriptive analyses of eye-level co-occurrence and phenotype characterisation.

### Outcomes, covariates and interaction analyses

The primary outcome was BCVA at Visit 4, measured in ETDRS letters. The primary prognostic analysis evaluated the association between baseline wdHRM and BCVA at V4. Secondary outcomes were the prevalence and short-term evolution of fibrosis-related and atrophy-related OCT biomarkers from baseline to V4, and their eye-level co-occurrence at Visit 4. Subretinal fluid (SRF) and intraretinal fluid (IRF) were analysed separately from fibrosis-related and atrophy-related OCT biomarkers. Prespecified covariates for the primary model were baseline BCVA, age, sex, ethnicity, MNV subtype, fluid status at V4, baseline (S)HRM, and baseline HTM. EZ- and ELM-loss were assessed in secondary descriptive analyses and were not included in the primary multivariable model. Analyses involving V4 pathology variables were restricted to eyes with available grading.

### Statistical analysis

Analyses were conducted at the eye level, with one eye included per patient. For the primary outcome, univariable linear regression models were first fitted for each prespecified predictor with BCVA at V4 as the dependent variable. A multivariable linear regression model was then used to estimate adjusted mean differences in BCVA at V4. Effect modification by MNV subtype was assessed by including wdHRM × MNV subtype interaction terms. Where interaction terms were statistically significant, estimated marginal means and simple contrasts were used to quantify adjusted differences in BCVA at V4 within each subtype. Descriptive statistics summarised baseline and V4 characteristics overall and across selected biomarker-defined groups. For descriptive two-group comparisons, continuous variables were compared using two-sample t tests and categorical variables using Pearson chi-square tests.

## Results

### Study cohort demographics and clinical characteristics

We analysed 2036 treatment-naïve eyes with exudative nAMD that completed three-dose aflibercept induction (median 112 days [IQR 98-119] from the first injection to Visit 4 [V4]). Mean baseline age was 79.4 years (SD 7.8); 60.8% of patients were female and 95.3% were White (Supplementary Table 1). Mean BCVA improved by 4.6 letters, from 58.0 (SD 14.5) at baseline to 62.6 (SD 15.2) at V4. The prevalence of SRF and IRF decreased from 83.0% and 50.8% at baseline to 37.6% and 21.7% at V4, respectively. At V4, 49.7% of eyes showed complete resolution of both SRF and IRF.

### Baseline predictors of BCVA at visit 4

**Fig. 3:**
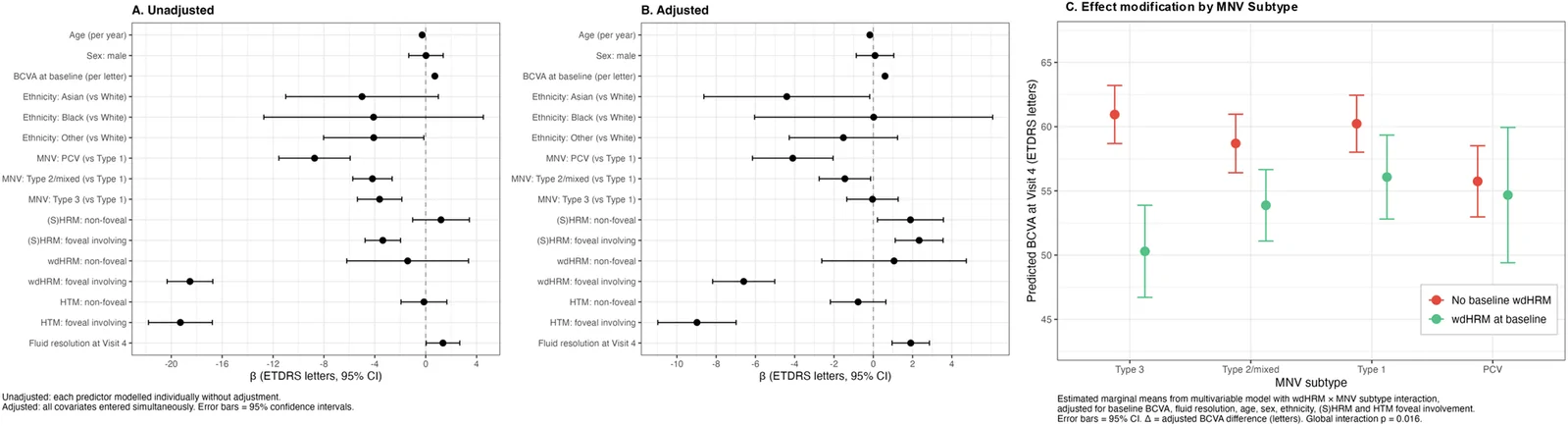
Baseline Predictors and Effect Modification of Best-Corrected Visual Acuity at Visit 4. **A, B** Forest plots showing regression coefficients (β) with 95% confidence intervals for associations between prespecified clinical and optical coherence tomography biomarkers and best-corrected visual acuity (BCVA), measured in Early Treatment Diabetic Retinopathy Study letters, at Visit 4 in 2036 treatment-naïve eyes with neovascular age-related macular degeneration from the PRECISE cohort. **A** Univariable linear regression models, in which each predictor was modelled individually without adjustment. **B** Multivariable linear regression model including baseline BCVA, age, sex, ethnicity, macular neovascularisation (MNV) subtype, baseline subretinal hyperreflective material (SHRM) category, baseline choroidal hypertransmission (HTM) category, baseline well-delineated hyperreflective material (wdHRM) category, and complete fluid resolution at Visit 4. The dashed vertical line indicates the null value (β = 0). Positive coefficients indicate higher BCVA at Visit 4, and negative coefficients indicate lower BCVA at Visit 4. Reference categories were female sex, White ethnicity, Type 1 MNV, absence of baseline SHRM, absence of baseline HTM, absence of baseline wdHRM, and persistent fluid at Visit 4. Model specification and the prespecified covariates are described in the *Methods* and Table 1. **C Predicted Best-Corrected Visual Acuity At Visit 4 According to Baseline Well-Delineated Hyperreflective Material Status across Macular Neovascularisation Subtypes**. Points represent estimated marginal means of best-corrected visual acuity (BCVA) at Visit 4, expressed in Early Treatment Diabetic Retinopathy Study letters, according to macular neovascularisation (MNV) subtype and baseline presence or absence of well-delineated hyperreflective material (wdHRM). Error bars indicate 95% confidence intervals. Estimates were derived from the secondary multivariable model including a baseline wdHRM × MNV subtype interaction term, adjusted for baseline BCVA, fluid resolution at Visit 4, age, sex, ethnicity, baseline SHRM category, and baseline hypertransmission category. Global interaction *p* = 0.016.

The primary multivariable model evaluated baseline wdHRM as the principal fibrosis-related biomarker of interest, with baseline HTM included as the atrophy-related adjustment biomarker; EZ- and ELM loss were examined separately in secondary descriptive analyses. In univariable linear regression models, baseline foveal-involving wdHRM was associated with 18.53 letters lower BCVA at V4 (95% CI, −20.32 to −16.73; *p* < 0.001). Foveal-involving baseline HTM was also strongly associated with lower V4 BCVA (β = −19.28; 95% CI, −21.79 to −16.77; *p* < 0.001), as was foveal-involving baseline (S)HRM (β = −3.37; 95% CI, −4.77 to −1.98; *p* < 0.001). Older age was associated with lower V4 BCVA (β = −0.28 letters per year; 95% CI, −0.37 to −0.20; *p* < 0.001). Compared with Type 1 MNV, PCV, Type 2/mixed MNV, and Type 3 MNV were each associated with lower V4 BCVA in unadjusted analyses. Baseline BCVA was strongly associated with V4 BCVA (β = 0.72 per letter; 95% CI, 0.68 to 0.75; *p* < 0.001), whereas sex, non-foveal baseline wdHRM, non-foveal baseline (S)HRM, and non-foveal baseline HTM were not associated with the outcome in these unadjusted models (Fig. 3A*;* Table 1).

**Table 1:** Unadjusted and Adjusted Regression Coefficients for Baseline Predictors of Best-Corrected Visual Acuity at Visit 4.

		Unadjusted		Adjusted	
Predictor	Level	β (95% CI)	*p*	β (95% CI)	*p*
**Age**	Age	*-0.28 (-0.37 to -0.20)*	*< 0.001*	*-0.17 (-0.23 to -0.11)*	*< 0.001*
**Sex**	Female (Ref)	*Reference*		*Reference*	
	Male	*0.02 (-1.34 to 1.37)*	*0.98*	*0.09 (-0.87 to 1.05)*	*0.85*
**BCVA at Baseline**	BCVA at Baseline	*0.72 (0.68 to 0.75)*	*< 0.001*	*0.60 (0.56 to 0.64)*	*< 0.001*
**Ethnicity**	White (Ref)	*Reference*		*Reference*	
	Asian	*-5.01 (-11.00 to 0.99)*	*0.10*	*-4.40 (-8.63 to -0.18)*	*0.04*
	Black	*-4.10 (-12.73 to 4.53)*	*0.35*	*0.02 (-6.04 to 6.09)*	*0.99*
	Other	*-4.09 (-8.02 to -0.15)*	*0.04*	*-1.52 (-4.28 to 1.24)*	*0.28*
**MNV Subtype**	Type 1 (Ref)	*Reference*		*Reference*	
	Type 2 and Mixed Types	*-4.19 (-5.73 to -2.64)*	*< 0.001*	*-1.44 (-2.75 to -0.13)*	*0.03*
	Type 3	*-3.62 (-5.37 to -1.88)*	*< 0.001*	*-0.04 (-1.35 to 1.27)*	*0.95*
	PCV	*-8.74 (-11.54 to -5.93)*	*< 0.001*	*-4.10 (-6.15 to -2.05)*	*< 0.001*
**(S)HRM at Baseline**	No (Ref)	*Reference*		*Reference*	
	Non-Foveal	*1.20 (-1.03 to 3.43)*	*0.29*	*1.89 (0.21 to 3.57)*	*0.03*
	Foveal-involving	*-3.37 (-4.77 to -1.98)*	*< 0.001*	*2.34 (1.12 to 3.56)*	*< 0.001*
**wdHRM at Baseline**	No (Ref)	*Reference*		*Reference*	
	Non-Foveal	*-1.42 (-6.20 to 3.36)*	*0.56*	*1.06 (-2.62 to 4.74)*	*0.57*
	Foveal-involving	*-18.53 (-20.32 to -16.73)*	*< 0.001*	*-6.60 (-8.18 to -5.02)*	*< 0.001*
**HTM at Baseline**	No (Ref)	*Reference*		*Reference*	
	Non-Foveal	*-0.14 (-1.94 to 1.66)*	*0.88*	*-0.77 (-2.19 to 0.64)*	*0.28*
	Foveal-involving	*-19.28 (-21.79 to -16.77)*	*< 0.001*	*-8.98 (-10.97 to -6.98)*	*< 0.001*
**Fluid Resolution at Visit 4**	Not Dry (Ref)	*Reference*		*Reference*	
	Dry	*1.35 (0.03 to 2.67)*	*0.04*	*1.91 (0.96 to 2.86)*	*< 0.001*
**wdHRM at Baseline × MNV Subtype**	wdHRM at Baseline × PCV	*-*	*-*	*3.08 (-2.71 to 8.88)*	*0.30*
	wdHRM at Baseline × Type 2 and Mixed Types	*-*	*-*	*-0.66 (-3.89 to 2.57)*	*0.69*
	wdHRM at Baseline × Type 3	*-*	*-*	*-6.50 (-10.50 to -2.50)*	*< 0.01*

*BCVA* Best-corrected visual acuity, *MNV* Macular neovascularisation, *PCV* Polypoidal Choroidal Vasculopathy, *(S)HRM* Subretinal hyperreflective Material, *wdHRM* Well-delineated hyperreflective material, *HTM* Choroidal Hypertransmission.

In the multivariable model including baseline BCVA, baseline wdHRM, MNV subtype, age, sex, ethnicity, baseline (S)HRM, baseline HTM, and fluid resolution at V4, baseline foveal-involving wdHRM remained independently associated with lower BCVA at V4 (β = −6.60; 95% CI, −8.18 to −5.02; *p* < 0.001). Foveal-involving baseline HTM retained the largest adjusted association with lower V4 BCVA (β = −8.98; 95% CI, −10.97 to −6.98; *p* < 0.001), whereas complete fluid resolution at V4 was independently associated with higher V4 BCVA (β = 1.91; 95% CI, 0.96 to 2.86; *p* < 0.001). Older age (β = −0.17 letters per year; 95% CI, −0.23 to −0.11; *p* < 0.001), PCV (β = −4.10; 95% CI, −6.15 to −2.05; *p* < 0.001), and Type 2 and mixed MNV (β = −1.44; 95% CI, −2.75 to −0.13; *p* = 0.03) also remained associated with lower V4 BCVA after adjustment, whereas Type 3 MNV was no longer significantly different from Type 1. In contrast to the unadjusted analyses, both foveal-involving and non-foveal baseline (S)HRM were associated with higher adjusted V4 BCVA, while non-foveal baseline wdHRM and non-foveal baseline HTM were not significantly associated with the outcome (Fig. 3B*;* Table 1).

### Visual outcomes stratified by MNV subtype

At baseline, neovascular subtype distribution was 39.2% for Type 1 MNV, 32.8% for Type 2 and mixed MNV, 21.7% for Type 3 MNV, and 6.3% for PCV. Baseline OCT and fluid profiles differed across subtypes (Table 2). Baseline wdHRM was most frequent in Type 2 and mixed MNV (23.1%) and PCV (14.8%), and less common in Type 3 MNV (12.2%) and Type 1 MNV (8.9%). By V4, wdHRM remained most frequent in Type 2 and mixed MNV (27.4%) and PCV (30.5%), compared with Type 3 MNV (19.5%) and Type 1 MNV (15.0%). Type 3 MNV showed the highest baseline prevalence of HTM (38.9%), followed by PCV (20.3%), Type 1 MNV (18.4%), and Type 2 and mixed MNV (12.7%). Baseline fluid phenotype also differed markedly by subtype. Type 3 MNV showed near-universal baseline IRF (99.3%) and comparatively low SRF prevalence (46.6%), whereas PCV showed the opposite pattern, with near-universal baseline SRF (99.2%) and lower IRF prevalence (42.2%). Type 1 and Type 2 and mixed MNV showed intermediate profiles, with high SRF prevalence and lower IRF prevalence. By V4, complete fluid resolution, defined as absence of both IRF and SRF, was most frequent in Type 3 MNV (66.1%) and least frequent in PCV (37.5%).

**Table 2:** Structural and Functional Outcomes Stratified by Macular Neovascularisation Subtype.

	PCV (*N* = 128)	Type 1 MNV (*N* = 799)	Type 2/Mixed Type MNV (*N* = 667)	Type 3 MNV (*N* = 442)	Overall (*N* = 2036)
**BCVA at Baseline**					
Mean (SD)	*53.0 (14.9)*	*61.6 (13.2)*	*55.6 (15.0)*	*56.6 (14.5)*	*58.0 (14.5)*
Median [Min, Max]	*54.0 [5.00,83.0]*	*65.0 [0,94.0]*	*58.0 [0, 87.0]*	*59.0 [0, 89.0]*	*60.0 [0, 94.0]*
**BCVA at Visit 4**					
Mean (SD)	*56.5 (17.4)*	*65.3 (13.8)*	*61.1 (15.5)*	*61.6 (15.8)*	*62.6 (15.2)*
Median [Min, Max]	*58.5 [0, 86.0]*	*69.0 [0,95.0]*	*65.0 [3.00, 87.0]*	*66.0 [0, 87.0]*	*66.0 [0, 95.0]*
**wdHRM at Baseline**					
Yes	*19 (14.8%)*	*71 (8.9%)*	*154 (23.1%)*	*54 (12.2%)*	*298 (14.6%)*
No	*109 (85.2%)*	*728 (91.1%)*	*513 (76.9%)*	*388 (87.8%)*	*1738 (85.4%)*
**wdHRM at Visit 4**					
Yes	*39 (30.5%)*	*120 (15.0%)*	*183 (27.4%)*	*86 (19.5%)*	*428 (21.0%)*
No	*88 (68.8%)*	*679 (85.0%)*	*478 (71.7%)*	*355 (80.3%)*	*1600 (78.6%)*
Missing	*1 (0.8%)*	*0 (0%)*	*6 (0.9%)*	*1 (0.2%)*	*8 (0.4%)*
**HTM at Baseline**					
No	*102 (79.7%)*	*652 (81.6%)*	*582 (87.3%)*	*270 (61.1%)*	*1606 (78.9%)*
Yes	*26 (20.3%)*	*147 (18.4%)*	*85 (12.7%)*	*172 (38.9%)*	*430 (21.1%)*
**HTM at Visit 4**					
No	*19 (14.8%)*	*277 (34.7%)*	*152 (22.8%)*	*70 (15.8%)*	*518 (25.4%)*
Yes	*108 (84.4%)*	*522 (65.3%)*	*509 (76.3%)*	*371 (83.9%)*	*1510 (74.2%)*
Missing	*1 (0.8%)*	*0 (0%)*	*6 (0.9%)*	*1 (0.2%)*	*8 (0.4%)*
**IRF at Baseline**					
Yes	*54 (42.2%)*	*229 (28.7%)*	*313 (46.9%)*	*439 (99.3%)*	*1035 (50.8%)*
No	*74 (57.8%)*	*570 (71.3%)*	*354 (53.1%)*	*3 (0.7%)*	*1001 (49.2%)*
**IRF at Visit 4**					
Yes	*36 (28.1%)*	*115 (14.4%)*	*157 (23.5%)*	*134 (30.3%)*	*442 (21.7%)*
No	*92 (71.9%)*	*684 (85.6%)*	*510 (76.5%)*	*308 (69.7%)*	*1594 (78.3%)*
**SRF at Baseline**					
Yes	*127 (99.2%)*	*744 (93.1%)*	*613 (91.9%)*	*206 (46.6%)*	*1690 (83.0%)*
No	*1 (0.8%)*	*55 (6.9%)*	*54 (8.1%)*	*236 (53.4%)*	*346 (17.0%)*
**SRF at Visit 4**					
Yes	*64 (50.0%)*	*389 (48.7%)*	*263 (39.4%)*	*50 (11.3%)*	*766 (37.6%)*
No	*64 (50.0%)*	*410 (51.3%)*	*404 (60.6%)*	*392 (88.7%)*	*1270 (62.4%)*
**Fluid Resolution at Visit 4**					
Dry	*48 (37.5%)*	*352 (44.1%)*	*320 (48.0%)*	*292 (66.1%)*	*1012 (49.7%)*
Not dry	*80 (62.5%)*	*447 (55.9%)*	*347 (52.0%)*	*150 (33.9%)*	*1024 (50.3%)*

*MNV* Macular neovascularisation, *PVC* Polypoidal choroidal Vasculopathy, *BCVA* Best-corrected visual acuity, *wdHRM* Well-delineated hyperreflective material, *HTM* Choroidal Hypertransmission, *IRF* Intraretinal fluid, *SRF* Subretinal fluid.

To assess whether the association between baseline wdHRM and V4 BCVA differed by subtype, the multivariable model was extended to include a wdHRM × MNV subtype interaction term. The global interaction was significant (*p* = 0.016), indicating that the adverse association of baseline wdHRM with V4 BCVA was not uniform across subtypes. Estimated marginal mean BCVA at V4 from the fully adjusted interaction model was 50.3 versus 60.9 letters in eyes with versus without baseline wdHRM in Type 3 MNV, 53.9 versus 58.7 letters in Type 2 and mixed MNV, 56.1 versus 60.2 letters in Type 1 MNV, and 54.7 versus 55.7 letters in PCV, corresponding to adjusted differences of −10.6, −4.8, −4.1, and −1.0 letters, respectively (Fig. 3C). Relative to the Type 1 reference, the interaction term was significantly negative for Type 3 MNV (β = −6.50; 95% CI, −10.50 to −2.50), whereas the interaction terms for Type 2 and mixed MNV (β = −0.66; 95% CI, −3.89 to 2.57) and PCV (β = 3.08; 95% CI, −2.71 to 8.88) were not significantly different from Type 1 (Table 1*)*.

### Evolution of fibrosis-related OCT biomarkers across therapy induction

At baseline, wdHRM was present in 14.6% of eyes and (S)HRM in 58.3%, most commonly as foveal-involving (S)HRM (47.3%). By V4, wdHRM prevalence had increased to 21.0%. Following induction, 7.3% of eyes had persistent wdHRM at both visits, 7.1% showed resolution of baseline wdHRM, 13.7% developed incident wdHRM despite being wdHRM-free at baseline, and 71.7% remained wdHRM-free at both visits. Eyes that developed or retained wdHRM by V4 already showed greater baseline HRM burden, including more frequent baseline (S)HRM and greater baseline HRM width and height (all *p* ≤ 0.001; Supplementary Table 2).

### Eye-level co-occurrence of fibrosis-related and atrophy-related OCT biomarkers

At baseline, atrophy-related OCT biomarkers were common but frequently limited by gradability. Amongst eyes with gradable HTM and wdHRM at both baseline and V4 (2028/2036 eyes; Supplementary Table 1), EZ and ELM were ungradable in 41.7% (845/2028) and 29.0% (589/2028) of eyes (Supplementary Table 2), respectively, reflecting lesion burden and scan limitations. Fibrosis-related OCT biomarkers were strongly associated with atrophy-related OCT biomarkers at the eye level. Among eyes with gradable wdHRM status, those with wdHRM at V4 had worse BCVA at baseline and at V4 than eyes without wdHRM (52 vs. 60 letters at baseline; 56 vs. 64 letters at V4; *p* < 0.001) (Supplementary Table 2). Fovea-involving EZ loss and ELM loss at baseline were approximately twice as frequent in eyes with wdHRM at V4, and HTM at V4 was more frequent in eyes with wdHRM than in those without wdHRM (95.6% [409/428] vs. 68.8% [1101/1600]; *p* < 0.001), whereas baseline HTM prevalence was similar between groups (*p* = 0.15). Conversely, among eyes with gradable HTM status, those with HTM at V4 had lower BCVA at baseline and V4 than eyes without HTM (56 vs. 63 letters at baseline; 61 vs. 68 letters at V4; *p* < 0.001), showed higher rates of baseline EZ and ELM loss, and were more likely to have baseline wdHRM (18.2% [275/1510] vs. 3.7% [19/518]; *p* < 0.001) and wdHRM at V4 (27.1% [409/1510] vs. 3.7% [19/518]; *p* < 0.001). (see Supplementary Table 3).

## Discussion

In this treatment-naïve nAMD cohort, baseline wdHRM was independently associated with poorer short-term BCVA after therapy induction, even after adjustment for baseline BCVA and prespecified covariates. Complete fluid resolution at V4 was independently associated with better BCVA, whereas the adverse association of baseline wdHRM differed across MNV subtypes. However, this early post-induction signal should not be equated with long-term visual prognosis, which will also depend on subsequent fibrosis progression, atrophic remodelling, recurrent disease activity, and cumulative treatment exposure.

The visual association of baseline wdHRM differed substantially across MNV subtypes, with the largest adjusted BCVA reduction observed in Type 3 MNV, moderate reductions in Type 1 and Type 2 and mixed lesions, and negligible short-term effect in PCV. This pattern is notable because Type 2 and mixed lesions carry higher cumulative fibrosis incidence over longer follow-up, indicating that early visual burden and long-term fibrosis propensity are not identical constructs [20]. In PRECISE, Type 3 eyes had the highest prevalence of baseline atrophy-related biomarkers and the strongest association between baseline fibrosis-related biomarkers and outer retinal disruption. These findings suggest that, particularly in Type 3 MNV, baseline wdHRM may capture broader fibro-atrophic remodelling rather than fibrosis alone.

### Fibro-atrophic remodelling rather than pure fibrosis

The strong eye-level association between fibrosis-related and atrophy-related OCT biomarkers in the secondary descriptive analyses suggests that wdHRM often reflects broader tissue remodelling rather than a single isolated histologic process [29, 30]. At V4, HTM was present in nearly all eyes with wdHRM, despite similar baseline HTM prevalence between groups, supporting the view that short-term evolution from baseline wdHRM involves fibro-atrophic remodelling rather than scar accumulation alone [19]. Because these analyses were descriptive and did not provide lesion-level spatial registration, they should be interpreted as phenotype-level association rather than proof that all atrophy-related biomarkers jointly contribute to the primary BCVA model. Importantly, our primary regression model adjusted for HTM as the atrophy-related biomarker available for that analysis, whereas EZ loss and ELM loss informed a separate secondary description of eye-level fibro-atrophic co-occurrence rather than the main prognostic model.

### Limitations

Several important limitations merit emphasis.

First, although baseline grading followed strict procedures, V4 regrading was performed without formal dual-grading. This asymmetry is expected to increase measurement error at V4 and, if misclassification is nondifferential, would bias associations toward the null, making effect sizes more likely to be underestimated than inflated.

Second, because HTM was graded from structural OCT as a binary biomarker rather than with *en face* registration or size quantification, some lesions may have been missed or underestimated, and we could not examine whether visual outcome varied according to HTM area or topographic extent.

Third, MNV subtype classification relied on structural OCT morphology without mandatory multimodal imaging at every visit, potentially biasing subtype-stratified analyses. Similarly, our operational definition also differs from the recent IFC consensus recommendations. The IFC workgroup proposed a stricter 2-step multimodal framework for detecting definite fibrosis in clinical studies, combining SD-OCT with confirmatory CFP or FA and favouring later post-treatment assessment, because OCT-only HRM in treatment-naïve eyes has limited specificity and may include non-fibrotic components. By contrast, PRECISE evaluated baseline wdHRM on structural OCT alone as a prognostic fibrosis-related biomarker in treatment-naïve eyes. Accordingly, our findings should be interpreted as relating to early OCT-defined fibro-atrophic remodelling and short-term visual prognosis, not as a multimodal adjudication of definite established fibrosis[14].

Finally, baseline wdHRM may partly reflect lesion chronicity or organisational stage at treatment initiation rather than tissue composition alone. Because PRECISE does not capture symptom duration or pre-presentation lesion age with sufficient precision, we cannot fully distinguish whether worse short-term visual outcomes in eyes with baseline wdHRM reflect more advanced fibrocellular organisation, delayed presentation, or both.

### Implications for research and practice

For clinical practice, the visual significance of baseline wdHRM should be interpreted in subtype context. In PCV, sub-RPE fibrovascular tissue may be compatible with limited short-term functional loss, whereas in Type 3 MNV the presence of baseline wdHRM appears to identify eyes at greater risk of limited early visual recovery after induction therapy. Prospective multimodal studies with spatial registration are needed to clarify lesion composition and the topographic relationship between fibrosis-related and atrophy-related biomarkers.

## Conclusion

In treatment-naïve nAMD, baseline wdHRM is associated with poorer short-term BCVA after induction therapy and with marked heterogeneity across MNV subtypes. Its strong eye-level association with atrophy-related OCT biomarkers in secondary analyses suggests that wdHRM often reflects broader fibro-atrophic remodelling rather than fibrosis alone. These findings support subtype-aware interpretation of early fibrosis-related OCT biomarkers and motivate prospective multimodal studies to clarify tissue composition and long-term prognostic relevance.

## Summary

### What was known before

- Subretinal fibrosis in neovascular AMD is associated with poorer visual outcomes, but OCT-defined hyperreflective material (HRM) encompasses both reversible exudation and fixed fibrotic tissue, complicating prognostication.
- Fibrosis risk and visual prognosis vary across macular neovascularisation (MNV) subtypes, yet early subtype-specific visual consequences of fibrosis-related OCT biomarkers during induction therapy remain poorly defined.

### What this study adds

- Baseline well-delineated hyperreflective material (wdHRM) independently predicts poorer short-term visual acuity after anti-VEGF induction, with the visual penalty paradoxically greater in eyes achieving complete fluid resolution.
- The visual impact of wdHRM is strongly modified by MNV subtype, being greatest in Type 3 MNV, moderate in Types 1 and 2/mixed, and negligible in polypoidal choroidal vasculopathy, supporting a subtype-specific fibro-atrophic phenotype.

## Supplementary information

Supplementary Table 1

Supplementary Table 2

Supplementary Table 3

## Data Availability

The anonymised PRECISE clinical database analysed during the current study is available from author SS on approval of a data sharing agreement with Moorfields Eye Hospital. Sharing of retinal images requires patient consent and sponsor approval.

## References

[1] Cheong KX, Cheung CMG, Teo KYC. Review of fibrosis in neovascular age-related macular degeneration. Am J Ophthalmol. 2023;246:192–222. 10.1016/j.ajo.2022.09.008 36162537

[2] Chakravarthy U, Harding SP, Rogers CA, Downes S, Lotery AJ, Dakin HA, et al. A randomised controlled trial to assess the clinical effectiveness and cost-effectiveness of alternative treatments to inhibit VEGF in Age-related choroidal Neovascularisation (IVAN). Health Technol Assess. 2015;19:1–298. 10.3310/hta19780 PMC4781416 26445075

[3] Daniel E, Pan W, Ying G-S, Kim BJ, Grunwald JE, Ferris FL 3rd, et al. Development and course of scars in the Comparison of Age-related Macular Degeneration Treatments Trials. Ophthalmology. 2018;125:1037–46. 10.1016/j.ophtha.2018.01.004 PMC6015772 29454660

[4] Daniel E, Ying G-S, Kim BJ, Toth CA, Ferris F 3rd, Martin DF, et al. Five-year follow-up of nonfibrotic scars in the Comparison of Age-Related Macular Degeneration Treatments Trials. Ophthalmology. 2019;126:743–51. 10.1016/j.ophtha.2018.11.020 PMC6475610 30476517

[5] Teo KYC, Joe AW, Nguyen V, Invernizzi A, Arnold JJ, Barthelmes D, et al. Prevalence and risk factors for the development of physician-graded subretinal fibrosis in eyes treated for neovascular age-related macular degeneration. Retina. 2020;40:2285–95. 10.1097/IAE.0000000000002779 32073543

[6] Gillies M, Arnold J, Bhandari S, Essex RW, Young S, Squirrell D, et al. Ten-year treatment outcomes of neovascular age-related macular degeneration from two regions. Am J Ophthalmol. 2020;210:116–24. 10.1016/j.ajo.2019.10.007 31606444

[7] Finger RP, Puth M-T, Schmid M, Barthelmes D, Guymer RH, Gillies M. Lifetime outcomes of anti-vascular endothelial growth factor treatment for neovascular age-related macular degeneration. JAMA Ophthalmol. 2020;138:1234–40. 10.1001/jamaophthalmol.2020.3989 PMC7563682 33057589

[8] Romano F, Cozzi E, Airaldi M, Nassisi M, Viola F, Aretti A, et al. Ten-year incidence of fibrosis and risk factors for its development in neovascular age-related macular degeneration. Am J Ophthalmol. 2023;252:170–81. 10.1016/j.ajo.2023.03.033 37030492

[9] Casalino G, Stevenson MR, Bandello F, Chakravarthy U. Tomographic biomarkers predicting progression to fibrosis in treated neovascular age-related macular degeneration: A multimodal imaging study. Ophthalmol Retin. 2018;2:451–61. 10.1016/j.oret.2017.08.019 31047325

[10] Nguyen CL, Gillies MC, Nguyen V, Daien V, Cohn A, Banerjee G, et al. Characterization of poor visual outcomes of neovascular age-related macular degeneration treated with anti-vascular endothelial growth factor agents. Ophthalmology. 2019;126:735–42. 10.1016/j.ophtha.2018.11.036 30529685

[11] Tenbrock L, Wolf J, Boneva S, Schlecht A, Agostini H, Wieghofer P, et al. Subretinal fibrosis in neovascular age-related macular degeneration: current concepts, therapeutic avenues, and future perspectives. Cell Tissue Res. 2022;387:361–75. 10.1007/s00441-021-03514-8 PMC8975778 34477966

[12] Bachmeier I, Armendariz BG, Yu S, Jäger RJ, Ebneter A, Glittenberg C, et al. Fibrosis in neovascular age-related macular degeneration: A review of definitions based on clinical imaging. Surv Ophthalmol. 2023;68:835–48. 10.1016/j.survophthal.2023.03.004 37023894

[13] Shah VP, Shah SA, Mrejen S, Freund KB. Subretinal hyperreflective exudation associated with neovascular age-related macular degeneration. Retina. 2014;34:1281–8. 10.1097/IAE.0000000000000166 24695062

[14] Chakravarthy U, Csincsik L, Teo KYC, Munk MR, Grewal DS, Guymer RH, et al. Standardization of Imaging Criteria for Detecting Macular Fibrosis in Neovascular Age-Related Macular Degeneration. Ophthalmology. Science. 2026;6:101027. 10.1016/j.xops.2025.101027 PMC12830330 41584096

[15] Willoughby AS, Ying G-S, Toth CA, Maguire MG, Burns RE, Grunwald JE, et al. Subretinal hyperreflective material in the Comparison of age-Related Macular Degeneration Treatments Trials. Ophthalmology. 2015;122:1846–53.e5. 10.1016/j.ophtha.2015.05.042 PMC4549177 26143666

[16] Roberts PK, Zotter S, Montuoro A, Pircher M, Baumann B, Ritter M, et al. Identification and quantification of the angiofibrotic switch in neovascular AMD. Invest Ophthalmol Vis Sci. 2019;60:304–11. 10.1167/iovs.18-25189 30657855

[17] Roberts PK, Schranz M, Motschi A, Desissaire S, Hacker V, Pircher M, et al. Morphologic and microvascular differences between macular neovascularization with and without subretinal fibrosis. Transl Vis Sci Technol. 2021;10:1. 10.1167/tvst.10.14.1 PMC8648059 34851359

[18] Armendariz BG, Chakravarthy U. Fibrosis in age-related neovascular macular degeneration in the anti-VEGF era. Eye. 2024;38:3243–51. 10.1038/s41433-024-03308-6 PMC11584703 39198703

[19] Lindenberg S, Nittala MG, Verma A, Fitzgerald MEC, Velaga SB, Bhisitkul RB, et al. Subretinal hyperreflective material in regions of atrophy and fibrosis in eyes with neovascular age-related macular degeneration. Can J Ophthalmol. 2025;60:26–34. 10.1016/j.jcjo.2024.05.007 38815954

[20] Lenhof S, Kodjikian L, Gascon P, Gadiollet E, Feldman A, De Bats F, et al. Subretinal fibrosis occurrence according to macular neovascularisation subtypes in neovascular age-related macular degeneration. Acta Ophthalmol. 2025;103:e104–e117. 10.1111/aos.16759 39319643

[21] Cheung CMG, Grewal DS, Teo KYC, Gan A, Mohla A, Chakravarthy U, et al. The evolution of fibrosis and atrophy and their relationship with visual outcomes in Asian persons with neovascular age-related macular degeneration. Ophthalmol. Retina. 2019;3:1045–55. 10.1016/j.oret.2019.06.002 31444144

[22] Chandak S, Gurudas S, Pakeer Muhammed R, Keskin A, Thottarath S, Ghanchi F, et al. Visual outcome following initiation of first injection versus after three monthly doses of aflibercept 2 mg for treatment naïve age-related macular degeneration to inform clinical trial designs: PRECISE Report No. 6. Eye. 2025;39:2194–203. 10.1038/s41433-025-03797-z PMC12274454 40355704

[23] Montesel A, Pakeer Muhammed R, Chandak S, Kazantzis D, Thottarath S, Chandra S, et al. Subretinal transient hyporeflectivity in neovascular age-related macular degeneration and its response to a loading phase of aflibercept: PRECISE report 4. Eye. 2024;38:2596–602. 10.1038/s41433-024-03087-0 PMC11385578 38653751

[24] Thottarath S, Gurudas S, Chandak S, Patel PJ, Kotagiri A, Pearce I, et al. Impact of treat and extend criteria on proportions that can be extended after loading phase of 2 mg aflibercept therapy for neovascular age related macular degeneration: PRECISE Report 5. Eye. 2024;38:2737–43. 10.1038/s41433-024-03110-4 PMC11427460 38710939

[25] Sivaprasad S, Chandak S, Gurudas S, Muhammed RP, Kazantzis D, Ghanchi F, et al. Factors associated with achieving various visual acuity outcomes during loading doses of aflibercept 2mg for treatment naïve exudative age-related macular degeneration: PRECISE Study Report 7. Res Square. 2024. Available at: 10.21203/rs.3.rs-5416918/v1 [Accessed October 28, 2025]. PMC12089498 39984700

[26] Chorev M, Haderlein J, Chandra S, Menon G, Burton BJL, Pearce I, et al. A multi-modal AI-driven cohort selection tool to predict suboptimal non-responders to aflibercept loading-phase for neovascular age-related macular degeneration: PRECISE. Study Rep 1 J Clin Med. 2023;12:3013. 10.3390/jcm12083013 PMC10142969 37109349

[27] Chandra S, Gurudas S, Burton BJL, Menon G, Pearce I, Mckibbin M, et al. Associations of presenting visual acuity with morphological changes on OCT in neovascular age-related macular degeneration: PRECISE Study Report 2. Eye. 2024;38:757–65. 10.1038/s41433-023-02769-5 PMC10920623 37853106

[28] Chandra S, Gurudas S, Pearce I, Mckibbin M, Kotagiri A, Menon G, et al. Baseline characteristics of eyes with early residual fluid post loading phase of aflibercept therapy in neovascular AMD: PRECISE study report 3. Eye. 2024;38:1301–7. 10.1038/s41433-023-02886-1 PMC11076629 38102473

[29] Teo KYC, Zhao J, Ibrahim FI, Fenner B, Chakravarthy U, Cheung CMG. Features associated with vision in eyes with subfoveal fibrosis from neovascular age-related macular degeneration. Am J Ophthalmol. 2024;261:121–31. 10.1016/j.ajo.2023.12.011 38135238

[30] Kim JH, Chang YS, Kim JW, Kim CG, Lee DW, Kim YJ. Morphologic features associated with fibrotic scarring after anti–vascular endothelial growth factor therapy in polypoidal choroidal vasculopathy. Retina. 2018;38:2168–76. 10.1097/IAE.0000000000001845 28930802

