# Supplementary material for: Macular neovascularisation subtype determines visual consequences of early fibro-atrophic remodelling in neovascular AMD: PRECISE study report 11
Source: Eye (Lond). 2026 Sep 17;40(16):2556–64. doi: 10.1038/s41433-026-04858-7 (PMC13755438; doi:10.1038/s41433-026-04858-7)
Supplement: Supplementary file 1 [file 41433_2026_4858_MOESM2_ESM.pdf]

	wdHRM at Visit 4 (N=428)	No wdHRM at Visit 4 (N=1600)	Overall at Visit 4 (N=2028)	wdHRM vs no wdHRM at Visit 4
**wdHRM at Baseline**				
Yes	149 (34.8%)	145 (9.1%)	294 (14.5%)	< 0.001
No	279 (65.2%)	1455 (90.9%)	1734 (85.5%)	
**BCVA at Baseline**				
Mean (SD)	52 (15)	60 (14)	58 (15)	< 0.001
Median (IQR)	53 (22)	62 (19)	60 (21)	
**BCVA at Visit 4**				
Mean (SD)	56 (17)	64 (14)	63 (15)	< 0.001
Median (IQR)	59 (24)	68 (16)	66 (19)	
**PED at Baseline**				
FVPED	316 (73.8%)	1209 (75.6%)	1525 (75.2%)	< 0.001
Haemorrhagic PED	33 (7.7%)	73 (4.6%)	106 (5.2%)	
No PED	35 (8.2%)	68 (4.3%)	103 (5.1%)	
Serous PED	44 (10.3%)	250 (15.6%)	294 (14.5%)	
**(S)HRM at Baseline**				
No	139 (32.5%)	707 (44.2%)	846 (41.7%)	< 0.001
Yes, Foveal	15 (3.5%)	70 (4.4%)	85 (4.2%)	
Yes, Foveal and Non-Foveal	225 (52.6%)	650 (40.6%)	875 (43.1%)	
Yes, Non-Foveal	49 (11.4%)	173 (10.8%)	222 (10.9%)	
**(S)HRM Width**				
Mean (SD)	1200 (1200)	810 (960)	880 (1000)	< 0.001
Median (IQR)	1000 (2000)	510 (1400)	580 (1600)	
**(S)HRM Height**				
Mean (SD)	130 (190)	91 (120)	100 (140)	< 0.001
Median (IQR)	120 (190)	82 (150)	92 (160)	
**IRF at Baseline**				
Yes	277 (64.7%)	753 (47.1%)	1030 (50.8%)	< 0.001
No	151 (35.3%)	847 (52.9%)	998 (49.2%)	
**SRF at Baseline**				
Yes	364 (85.0%)	1319 (82.4%)	1683 (83.0%)	0.23
No	64 (15.0%)	281 (17.6%)	345 (17.0%)	
**IRF at Visit 4**				
Yes	119 (27.8%)	319 (19.9%)	438 (21.6%)	< 0.001
No	309 (72.2%)	1281 (80.1%)	1590 (78.4%)	
**SRF at Visit 4**				
Yes	128 (29.9%)	634 (39.6%)	762 (37.6%)	< 0.001
No	300 (70.1%)	966 (60.4%)	1266 (62.4%)	
**Fluid Resolution at Visit 4**				
Dry	226 (52.8%)	784 (49.0%)	1010 (49.8%)	0.18
Not dry	202 (47.2%)	816 (51.0%)	1018 (50.2%)	
**EZ-loss at Baseline**				
No	65 (15.2%)	525 (32.8%)	590 (29.1%)	< 0.001
Ungradable	173 (40.4%)	672 (42.0%)	845 (41.7%)	
Yes, Foveal involving	146 (34.1%)	235 (14.7%)	381 (18.8%)	
Yes, Non-Foveal	44 (10.3%)	168 (10.5%)	212 (10.5%)	
**ELM-loss at Baseline**				
No	127 (29.7%)	769 (48.1%)	896 (44.2%)	< 0.001
Ungradable	125 (29.2%)	464 (29.0%)	589 (29.0%)	
Yes, Foveal involving	127 (29.7%)	199 (12.4%)	326 (16.1%)	
Yes, Non-Foveal	49 (11.4%)	168 (10.5%)	217 (10.7%)	
**HTM at Baseline**				
No	326 (76.2%)	1272 (79.5%)	1598 (78.8%)	0.15
Yes	102 (23.8%)	328 (20.5%)	430 (21.2%)	
**HTM at Visit 4**				
No	19 (4.4%)	499 (31.2%)	518 (25.5%)	< 0.001
Yes	409 (95.6%)	1101 (68.8%)	1510 (74.5%)	

wdHRM = Well-delineated hyperreflective material; BCVA = Best-corrected visual acuity; PED = Pigment epithelium detachment; FVPED = Fibrovascular PED; (S)HRM = (Subretinal) hyperreflective material; IRF = Intraretinal fluid; SRF = Subretinal fluid; EZ = Ellipsoid Zone; ELM = External limiting membrane; HTM = Hypertransmission

1
