# Supplementary material for: Macular neovascularisation subtype determines visual consequences of early fibro-atrophic remodelling in neovascular AMD: PRECISE study report 11
Source: Eye (Lond). 2026 Sep 17;40(16):2556–64. doi: 10.1038/s41433-026-04858-7 (PMC13755438; doi:10.1038/s41433-026-04858-7)
Supplement: Supplementary file 2 [file 41433_2026_4858_MOESM3_ESM.pdf]

	No HTM at Visit 4 (N=518)	HTM at Visit 4 (N=1510)	Overall (N=2028)	No HTM versus HTM at Visit 4
**HTM at Baseline**				
No	*483 (93.2%)*	*1115 (73.8%)*	*1598 (78.8%)*	*< 0.001*
Yes	*35 (6.8%)*	*395 (26.2%)*	*430 (21.2%)*	
**BCVA at Baseline**				
Mean (SD)	*63 (12)*	*56 (15)*	*58 (15)*	*< 0.001*
Median (IQR)	*65 (14)*	*58 (22)*	*60 (21)*	
**BCVA at Visit 4**				
Mean (SD)	*68 (11)*	*61 (16)*	*63 (15)*	*< 0.001*
Median (IQR)	*70 (12)*	*65 (21)*	*66 (19)*	
**PED at Baseline**				
FVPED	*412 (79.5%)*	*1113 (73.7%)*	*1525 (75.2%)*	*< 0.001*
Haemorrhagic PED	*12 (2.3%)*	*94 (6.2%)*	*106 (5.2%)*	
No PED	*16 (3.1%)*	*87 (5.8%)*	*103 (5.1%)*	
Serous PED	*78 (15.1%)*	*216 (14.3%)*	*294 (14.5%)*	
**(S)HRM at Baseline**				
No	*267 (51.5%)*	*579 (38.3%)*	*846 (41.7%)*	*< 0.001*
Yes, Foveal	*20 (3.9%)*	*65 (4.3%)*	*85 (4.2%)*	
Yes, Foveal and Non-Foveal	*169 (32.6%)*	*706 (46.8%)*	*875 (43.1%)*	
Yes, Non-Foveal	*62 (12.0%)*	*160 (10.6%)*	*222 (10.9%)*	
**(S)HRM Width**				
Mean (SD)	*590 (770)*	*990 (1100)*	*880 (1000)*	*< 0.001*
Median (IQR)	*0 (1000)*	*750 (1700)*	*580 (1600)*	
**(S)HRM Height**				
Mean (SD)	*79 (140)*	*110 (140)*	*100 (140)*	*< 0.001*
Median (IQR)	*0 (140)*	*100 (170)*	*92 (160)*	
**IRF at Baseline**				
Yes	*179 (34.6%)*	*851 (56.4%)*	*1030 (50.8%)*	*< 0.001*
No	*339 (65.4%)*	*659 (43.6%)*	*998 (49.2%)*	
**SRF at Baseline**				
Yes	*457 (88.2%)*	*1226 (81.2%)*	*1683 (83.0%)*	*< 0.001*
No	*61 (11.8%)*	*284 (18.8%)*	*345 (17.0%)*	
**EZ-loss at Baseline**				
No	*252 (48.6%)*	*338 (22.4%)*	*590 (29.1%)*	*< 0.001*
Ungradable	*214 (41.3%)*	*631 (41.8%)*	*845 (41.7%)*	
Yes, Foveal involving	*25 (4.8%)*	*356 (23.6%)*	*381 (18.8%)*	
Yes, Non-Foveal	*27 (5.2%)*	*185 (12.3%)*	*212 (10.5%)*	
**ELM-loss at Baseline**				
No	*367 (70.8%)*	*529 (35.0%)*	*896 (44.2%)*	*< 0.001*
Ungradable	*115 (22.2%)*	*474 (31.4%)*	*589 (29.0%)*	
Yes, Foveal involving	*14 (2.7%)*	*312 (20.7%)*	*326 (16.1%)*	
Yes, Non-Foveal	*22 (4.2%)*	*195 (12.9%)*	*217 (10.7%)*	
**wdHRM at Baseline**				
Yes	*19 (3.7%)*	*275 (18.2%)*	*294 (14.5%)*	*< 0.001*
No	*499 (96.3%)*	*1235 (81.8%)*	*1734 (85.5%)*	
**wdHRM at Visit 4**				
Yes	*19 (3.7%)*	*409 (27.1%)*	*428 (21.1%)*	*< 0.001*
No	*499 (96.3%)*	*1101 (72.9%)*	*1600 (78.9%)*	

wdHRM = Well-delineated hyperreflective material; BCVA = Best-corrected visual acuity; PED = Pigment epithelium detachment; (S)HRM = (Subretinal) hyperreflective material; IRF = Intraretinal fluid; SRF = Subretinal fluid; EZ = Ellipsoid Zone; ELM = External limiting membrane; HTM = Hypertransmission

* Percentages are column percentages within the displayed comparison groups, based on eyes with available grading for the relevant biomarker. Denominators therefore vary across rows because gradability differs by biomarker.
