# Supplementary material for: Macular neovascularisation subtype determines visual consequences of early fibro-atrophic remodelling in neovascular AMD: PRECISE study report 11
Source: Eye (Lond). 2026 Sep 17;40(16):2556–64. doi: 10.1038/s41433-026-04858-7 (PMC13755438; doi:10.1038/s41433-026-04858-7)
Supplement: Supplementary file 3 [file 41433_2026_4858_MOESM1_ESM.docx]

|  | Visit 1 (Baseline) (N=2036) | Visit 4 (N=2036) |
| --- | --- | --- |
| **Sex** |  |  |
| Female | *1237 (60.8%)* |  |
| Male | *799 (39.2%)* |  |
| **Age (Years)** | *79.4 (7.8)* |  |
| Median [IQR] | *80 [74 - 85]* |  |
| **Ethnicity** |  |  |
| White | *1940 (95.3%)* |  |
| Asian | *25 (1.2%)* |  |
| Black | *12 (0.6%)* |  |
| Other | *59 (2.9%)* |  |
| **BCVA (ETDRS Letters)** | *58.0 (14.5)* | *62.6 (15.2)* |
| Median [IQR] | *60 [49 - 70]* | *66 [55 - 74]* |
| **HTM** |  |  |
| No | *1606 (78.9%)* | *518 (25.4%)* |
| Non-Foveal | *292 (14.3%)* | *150 (7.4%)* |
| Foveal-involving | *138 (6.8%)* | *1306 (64.1%)* |
| Missing | *0 (0%)* | *62 (3.0%)* |
| **wdHRM** |  |  |
| No | 1738 (85.4%) | 1600 (78.6%) |
| Non-Foveal | 33 (1.6%) | 61 (3.0%) |
| Foveal-involving | 265 (13.0%) | 313 (15.4%) |
| Missing | 0 (0%) | 62 (3.0%) |
| **(S)HRM** |  |  |
| No | 849 (41.7%) | 1378 (67.7%) |
| Non-Foveal | 223 (11.0%) | 140 (6.9%) |
| Foveal-involving | 964 (47.3%) | 457 (22.4%) |
| Missing | 0 (0%) | 61 (3.0%) |
| **IRF** |  |  |
| Yes | *1035 (50.8%)* | *442 (21.7%)* |
| No | *1001 (49.2%)* | *1594 (78.3%)* |
| **SRF** |  |  |
| Yes | *1690 (83.0%)* | *766 (37.6%)* |
| No | *346 (17.0%)* | *1270 (62.4%)* |
| **Fluid Resolution** |  |  |
| Dry | *3 (0.1%)* | *1012 (49.7%)* |
| Not dry | *2033 (99.9%)* | *1024 (50.3%)* |
| **MNV Subtype** |  |  |
| PCV | *128 (6.3%)* |  |
| Type 1 | *799 (39.2%)* |  |
| Type 2 and Mixed Types | *667 (32.8%)* |  |
| Type 3 | *442 (21.7%)* |  |
| **PED Type** |  |  |
| Fibrovascular | *1533 (75.3%)* |  |
| Haemorrhagic | *106 (5.2%)* |  |
| No PED | *103 (5.1%)* |  |
| Serous | *294 (14.4%)* |  |
|  |  |  |
| BCVA = Best-corrected visual acuity; ETDRS = Early Treatment Diabetic Retinopathy Study; MNV = Macular neovascularisation; PED = Pigment epithelium detachment; wdHRM = Well-delineated hyperreflective material; IRF = Intraretinal fluid; SRF = Subretinal fluid; PCV = Polypoidal Choroidal Vasculopathy; HTM = Hypertransmission | | |
